# Plant-based dietary indices and biomarkers of chronic low-grade inflammation: a cross-sectional analysis of adults in Ireland

**DOI:** 10.1007/s00394-023-03242-5

**Published:** 2023-09-02

**Authors:** Soraeya Kharaty, Janas M. Harrington, Seán R. Millar, Ivan J. Perry, Catherine M. Phillips

**Affiliations:** 1 School of Public Health, Physiotherapy and Sports Science, University College Dublin, Dublin 4, Ireland; 2 School of Public Health, University College Cork, Cork, Ireland

**Keywords:** Plant-based diet, Plant-based dietary index, Middle-aged adults, Cardiovascular disease, Inflammatory biomarkers, Cross-sectional study

## Abstract

**Purpose:**

There is increasing interest in the health benefits of plant-based diets (PBDs). Evidence reports favourable associations with inflammatory profiles and reduced cardiovascular disease risk. However, limited studies have examined relationships between PBD indices (PDIs) and inflammatory biomarkers. We explored overall PDI, healthful PDI (hPDI) and unhealthful PDI (uPDI) associations with inflammatory biomarker profiles.

**Methods:**

This cross-sectional analysis included 1986 middle- to older-aged adults from the Mitchelstown Cohort. PDI scores were calculated using validated food frequency questionnaires. PDI score associations with inflammatory biomarkers were assessed via linear regression analysis, with adjustment for potential confounders.

**Results:**

Comparison of quintiles (Q5 vs Q1) revealed lower concentrations of C-reactive protein (CRP), interleukin 6 (IL-6), white blood cells (WBCs), neutrophils and monocytes, and the leptin-to-adiponectin ratio (PDI and hPDI *P* < 0.05); lower leptin (PDI, *P* < 0.05), and complement component 3 (C3), tumour necrosis factor alpha (TNF*-*α), plasminogen activator inhibitor 1, lymphocytes and eosinophils (hPDI, *P* < 0.05); and higher concentrations of adiponectin (PDI and hPDI, *P* < 0.05). Conversely, higher concentrations of C3, CRP, IL-6, TNF-α, resistin, WBCs, neutrophils, lymphocytes, monocytes and eosinophils, and the neutrophil-to-lymphocyte ratio, and lower adiponectin concentrations were observed comparing uPDI quintiles (*P* < 0.05). In fully adjusted regression models, higher hPDI scores were associated with lower concentrations of C3, TNF-*α,* WBCs, neutrophils and monocytes (all *P* < 0.01). Higher uPDI scores were associated with higher C3 and TNF-*α* concentrations (all *P* < 0.01).

**Conclusion:**

This study provides evidence that a more healthful PBD is associated with a more favourable inflammatory profile and that a more unhealthful PBD is associated with the reverse.

**Supplementary Information:**

The online version contains supplementary material available at 10.1007/s00394-023-03242-5.

## Introduction

Non-communicable diseases (NCDs), including cardiovascular disease (CVD), type 2 diabetes and cancers are leading causes of ill health and premature mortality worldwide [1]. A plant-based diet (PBD), defined as a dietary pattern in which foods of animal origin are totally or mostly excluded [2], has been recommended by the World Health Organization as part of a healthy lifestyle to prevent NCDs [3]. The recent healthy reference diet from the EAT-*Lancet* Commission also recommends a more PBD as an effective way to improve population and planetary health [4].

Evidence suggests that PBDs are associated with more favourable intermediate biomarkers of CVD risk and a lower risk of CVD events and mortality [5–7]. However, a number of previous studies have not considered the quality of plant-based foods. This is important, as not all plant-based foods are thought to have favourable cardiometabolic effects [8]. To overcome such limitations, PBD scores have been developed to differentiate between lower and higher quality PBDs [9]. The overall plant-based diet index (PDI) emphasises the consumption of plant-based foods and limits animal-based foods; the healthful plant-based diet index (hPDI) emphasises the consumption of healthy plant-based foods and limits unhealthy plant-based and animal-based foods; the unhealthful plant-based diet index (uPDI) emphasises the consumption of unhealthy plant-based foods and limits healthy plant-based and animal-based foods [9].

Higher adherence to the PDI and hPDI has been shown to be associated with a reduced risk of CVD events, CVD mortality and type 2 diabetes, while research has suggested higher adherence to the uPDI to be associated with a higher risk of these conditions [9–13]. Low-grade systemic inflammation may be a mediating factor as it is thought to play a role in the initiation and progression of atherosclerosis [14]. During atherogenesis, an innate immune response is initiated by endothelial cells, forming inflammatory cytokines such as interleukin-6 (IL-6) and tumour necrosis factor alpha (TNF-α) [15]. As chronic low-grade inflammation accompanies all stages of atherosclerotic disease, elevated levels of circulating inflammatory biomarkers may predict CVD risk [16].

A more pro-inflammatory diet, characterised by a higher Dietary Inflammatory Index^®^ (DII) score, has been linked with a higher risk of CVD and associated mortality [17]. There is also evidence, albeit not conclusive, to suggest that PBDs may have anti-inflammatory effects [5] and results from PDI and DII correlation analysis suggest that the PDI and hPDI are less inflammatory and the uPDI more inflammatory [18]. However, limited investigation of PDI associations with inflammatory biomarkers has been conducted [19, 20].

Examining relationships between PDIs and a range of inflammatory biomarkers is therefore necessary in order to gain a better understanding of the underlying mechanisms through which PBDs may influence cardiometabolic health. Therefore, the aim of this study was to investigate whether a PBD is associated with inflammation in a cohort of middle- to older-aged adults, and whether this relationship differs by PBD quality. In particular, we assessed relationships between adherence to the hPDI and uPDI and a range of inflammatory biomarkers to test the hypothesis that the hPDI would be associated with a more favourable, and the uPDI a less favourable, inflammatory biomarker profile.

## Methods

### Study population and setting

The Cork and Kerry Diabetes and Heart Disease Study (Mitchelstown Cohort Study–Phase II) was a cross-sectional study carried out between 2010 and 2011. The Livinghealth Clinic serves a local population of approximately 20000 white European individuals with a mix of urban and rural residents. Participants were randomly selected from all registered middle- to older-aged (46–73 years) attending patients, and a stratified sample was recruited. Of 3807 potential participants, following the exclusion of duplicates, deaths and subjects incapable of consenting or attending appointment, 3051 were invited to take part in the study. Of these, 2047 (49% male) completed the questionnaire and physical examinations of the baseline assessment (response of 67%). Participants were broadly similar to the local background population in terms of proportional age representation, marital status and education, representing a low risk of selection bias [21]. Of the 2047 individuals that completed baseline assessment, dietary data was available for 1986 participants (Supplemental Fig. 1). Ethics committee approval conforming to the Declaration of Helsinki was obtained from the Clinical Research Ethics Committee of University College Cork (reference ECM 4 (aa) 02/02/10). A letter was sent out by the general practitioner (GP) to all participants, with a reply slip indicating acceptance to the study. All participants provided signed informed consent to use their data for research purposes [22].

### Data collection

All participants completed a General Health Questionnaire (GHQ) which was used to collect demographic and health-related variables which included sex, age, level of education, smoking status, alcohol consumption, prescription medication use, multivitamin use and disease history. Information on frequency, duration and intensity of physical activity was collected and the validated International Physical Activity Questionnaire (IPAQ) was used to assess physical activity levels [23].

### Clinical measurements

Anthropometric measurements were performed by trained researchers with calibrated instruments using standard operating procedure manuals. Weight was measured in kilograms without shoes, to the nearest 100 g, using a calibrated portable electronic Tanita WB-100MA weighing scale (Tanita Corp., Arlington Heights, IL, USA). Height was measured in centimetres to 1 decimal place using a Seca Leicester height gauge (Seca, Birmingham, UK). Body mass index (BMI) was calculated as weight in kilograms divided by the square of height in meters. Three measurements of systolic and diastolic blood pressure were obtained with the subject in a seated position using an Omron M7 Digital sphygmomanometer (Omron Healthcare Co. Ltd., Japan). The mean of the second and third readings was considered as a subject’s blood pressure.

### Dietary assessment

A modified version of the self-completed European Prospective Investigation into Cancer and Nutrition food frequency questionnaire (FFQ), which has been validated in several populations, was used for dietary assessment [24]. The FFQ was originally validated in the Irish population using food diaries and a protein biomarker in a volunteer sample [25], and incorporated into the SLÁN Irish National Surveys of Lifestyle Attitudes and Nutrition 1998, 2002, 2007 [26–28]. The semi-quantitative FFQ was adapted to include 150 food items to reflect the Irish diet. Average medium servings of each food item consumed by participants over the last 12 months were converted into quantities using standard portion sizes. Quantity was expressed as grams per day for food items and millilitres per day for beverages. Daily energy and nutrient intakes were calculated from the data collected from the FFQ using a tailored computer program (FFQ Software Ver 1.0; developed by the National Nutrition Surveillance Centre, School of Public Health, Physiotherapy and Sports Science, University College Dublin, Belfield, Dublin 4, Ireland). This linked frequency selections with the food equivalents in McCance and Widdowson Food Tables [29].

### Plant-based diet indices

The PDIs were created using the method described by Satija et al. [9]. Food items from the FFQ were organised into 18 food groups. The food groups were then categorised into ‘healthy’ plant foods (whole grains, fruits, vegetables, nuts, legumes, vegetable oils and tea and coffee), ‘unhealthy’ plant foods (refined grains, potatoes, fruit juices, sugar-sweetened beverages (SSBs) and sweets and desserts) and animal foods (animal fats, dairy, eggs, meat, fish or seafood and miscellaneous animal-based foods). Margarine intake was included in the vegetable oil food group as *trans *fatty acid consumption was trivial in the Irish population at the time of data collection [30].

Frequencies of consumption of each food were converted into servings consumed per day. The number of servings of foods that belonged to each of the 18 food groups were then totalled. Each of the 18 food groups were divided into quintiles of consumption and were assigned a score from 1 to 5 [9]. However, due to the skewed distribution of consumption, nuts were divided into tertiles of consumption and assigned a score from 1 to 3, and SSBs and eggs were divided into quartiles of consumption and assigned a score from 1 to 4.

For the overall PDI, the quintile with the highest consumption of plant-based foods and the lowest consumption of animal-based foods was assigned a score of 5 (3 for nuts; 4 for SSBs and eggs) and quintiles with the lowest consumption of plant-based foods and the highest consumption of animal-based foods were assigned a score of 1. For the hPDI, only healthy plant foods received positive scores. Participants with the highest consumption of healthy plant-based foods received a score of 5 while participants with the highest consumption of unhealthy plant-based foods and animal-based foods received a score of 1. For the uPDI, only unhealthy plant foods received positive scores. Participants with the highest consumption of unhealthy plant-based foods received a score of 5 while participants with the highest consumption of healthy plant-based foods and animal-based foods received a score of 1 [9].

The food groups were summarised to generate PDI, hPDI and uPDI scores. A higher PDI score indicates a more plant-based and less animal-based diet, a higher hPDI score indicates a more healthful PBD and a less unhealthful and animal-based diet and a higher uPDI score indicates a more unhealthful PBD and a less healthful and animal-based diet. The PDI, hPDI and uPDI indices had a theoretical range of 18–86, where 18 represents lowest adherence to the index and 86 represents greatest adherence to the index. Missing data for food group intake were treated as zero consumption because our experience was that individuals most often left food group items ‘blank’ when they did not habitually eat the food item, a pattern also demonstrated in the Nurses’ Health Study (NHS) II and the Health Professionals Follow-Up Study (HPFS) [31]. In this sample the ranges for each score were 31–72 for the PDI, 31–76 for the hPDI and 30–75 for the uPDI.

### Inflammatory profiling

Fasting (minimum 8 h) blood samples were taken from all participants in the morning on arrival to the clinic. Plasma and serum were prepared for biological analysis as previously described [32]. Serum C-reactive protein (CRP), TNF-α, IL-6, adiponectin, leptin, resistin and plasminogen activator inhibitor 1 (PAI-1) were assessed using a biochip array system (Evidence Investigator; Randox Laboratories). Complement component 3 (C3) was measured by immunoturbidimetric assay (Rx Daytona; Randox Laboratories). Total white blood cell (WBC) count, neutrophil, lymphocyte, monocyte, eosinophil and basophil concentrations were determined by flow cytometry technology by the Cork University Hospital Haematology Laboratory using fresh blood samples. The neutrophil-to-lymphocyte ratio (NLR) was calculated as neutrophils divided by lymphocytes. The leptin-to-adiponectin ratio (LAR) was calculated as leptin divided by adiponectin. Serum glycoprotein A (glycA) was measured on serum specimens by nuclear magnetic resonance spectroscopy (*NMR LipoProfile*^®^ analysis) at LipoScience Inc (Raleigh, NC, USA) [33].

### Classification of variables

Based on the literature, potential confounding variables include sex, age (years), level of education, smoking status, alcohol consumption, physical activity, anti-inflammatory medication use, multivitamin use, type 2 diabetes status, CVD status, hypertension, BMI and energy intake (kcal). Categories of education included: primary education (finished full-time education at age 13 years or younger, some primary or primary or equivalent), secondary education (intermediate/group certificate or equivalent or leaving certificate or equivalent) and tertiary education (diploma/certificate, primary university degree or postgraduate/higher degree). Smoking status was categorised as current smoker (smoking at present) and never/former smoker (having never smoked at least 100 cigarettes in their life/having smoked 100 cigarettes in their life but did not smoke at present). Alcohol consumption included questions regarding past and current intake (measured in units of alcohol consumed on a weekly basis). Alcohol consumption was categorised as non-drinker (< 1 drink per week), moderate drinker (between 1 and 14 drinks per week) and heavy drinker (> 14 drinks per week). Physical activity was categorised as low, moderate and high levels of activity using the IPAQ. Type 2 diabetes was defined according to the American Diabetes Association guidelines as a fasting plasma glucose level ≥ 7 mmol/L or a haemoglobin A1C level ≥ 6.5% (≥ 48 mmol/mol) or by a physician diagnosis [34]. The presence of CVD was obtained from the GHQ by asking study participants if they had been diagnosed with one of the following seven conditions: heart attack (including coronary thrombosis or myocardial infarction (MI)), heart failure, angina, aortic aneurysm, hardening of the arteries, stroke or any other heart trouble. Subjects indicating a diagnosis of any of these disorders were classified as having CVD. Hypertension was defined as a systolic blood pressure reading ≥ 140 mmHg and/or a diastolic blood pressure reading ≥ 90 mmHg [35].

## Statistical analysis

Statistical analysis was conducted using SPSS Version 27 for MacOS (IBM Corporation, Armonk, NY, USA). Normality was assessed by visual inspection of variable distribution using histograms and using the Shapiro–Wilk test, where a significant result (*P* ≤ 0.05) suggested a violation of normality. Continuous variables are expressed as means ± one standard deviation, categorical variables as percentages and a median and interquartile range are shown for skewed data. Differences were analysed using an independent *t* test or a Mann–Whitney *U* test for continuous variables and a Pearson’s chi-square test for categorical variables. Characteristics of the study population were compared according to the lowest and highest PDI, hPDI and uPDI quintiles. Correlations between dietary scores and dietary and nutrient intakes were examined using Spearman’s rank-order correlation (Supplemental Table 1). Linear regression was used to determine PDI, hPDI and uPDI score associations with inflammatory biomarkers. The distribution of residuals was examined for normality via visual inspection of P–P plots and skewed biomarker data were log-transformed for linear regression analysis. Three models were run for the main analysis: model one tested crude associations; a second model was adjusted for sex and age and a third fully adjusted model additionally adjusted for education (primary, secondary and tertiary), smoking status (never/former and current), alcohol consumption (none, moderate and heavy), physical activity (low, moderate and high), anti-inflammatory medication use, multivitamin use, type 2 diabetes status, CVD status, hypertension or anti-hypertensive medication use, BMI and energy intake. A fourth model was run with the fully adjusted model excluding BMI and energy intake, as a sensitivity analysis.

A restricted analysis was conducted excluding participants with implausible energy intakes to avoid extreme misreporting by considering sex-specific cut-offs of < 800 and > 4000 kcal/d for males (*n* = 39) and < 500 and > 3500 kcal/d for females (*n* = 62) (Supplemental Table 2) [31]. Another sensitivity analysis was conducted with boiled/mashed potatoes scored positively for the hPDI, and negatively for the uPDI, by adding these to the ‘vegetables’ food group (Supplemental Tables 3 and 4). Contingency tables comparing the proportion of individuals in quintiles of the PDI, hPDI and uPDI are presented in Supplemental Tables 5, 6 and 7. For all analyses, a *P* value (two-tailed) of less than or equal to 0.05 was considered to indicate statistical significance. To correct for the multiple testing performed in the linear regression analysis and reduce the risk of Type I errors, false discovery rate adjusted *P* values were calculated using the method described by Benjamini and Hochberg (data not shown) [36].

## Results

### Descriptive characteristics

Characteristics of the study population for the full sample and according to sex are presented in Table 1**.** Significant differences between males and females were noted for education, alcohol consumption, physical activity levels, anti-inflammatory medication and multivitamin use, prevalence of type 2 diabetes and CVD, BMI and for the PDI, hPDI and uPDI scores, with female participants having higher PDI and hPDI scores and lower uPDI scores, compared to males. Sex differences were also observed for all inflammatory biomarkers except for lymphocyte and basophil concentrations.

**Table 1:** Characteristics of the study population—full sample and stratified by sex

Variable	Full sample (*n* = 1986)	Males (*n* = 972)	Females (*n* = 1014)	*P*
General				
Age, years [median (IQR)]	59.5 (55.0–64.1)	59.3 (55.0–64.2)	59.6 (55.0–64.0)	0.833^c^
Primary education (%)	520 (27.8)	297 (32.1)	223 (23.6)	< 0.001^b^
Secondary education (%)	917 (49.0)	445 (48.1)	472 (49.9)	0.44^b^
Tertiary education (%)	434 (23.2)	183 (19.8)	251 (26.5)	< 0.001^b^
Current smoker (%)	283 (14.6)	139 (14.7)	144 (14.5)	0.919^b^
Non-drinker (%)	209 (16.0)	103 (14.4)	106 (18.0)	0.079^b^
Moderate drinker (%)	883 (67.6)	421 (58.8)	462 (78.3)	< 0.001^b^
Heavy drinker (%)	214 (16.4)	192 (26.8)	22 (3.7)	< 0.001^b^
Low-level physical activity (%)	905 (48.1)	381 (42.2)	524 (53.6)	< 0.001^b^
Moderate-level physical activity (%)	561 (29.8)	255 (28.3)	306 (31.3)	0.153^b^
High-level physical activity (%)	414 (22.0)	266 (29.5)	148 (15.1)	< 0.001^b^
Anti-inflammatory medication use (%)	391 (19.7)	226 (23.3)	165 (16.3)	< 0.001^b^
Multivitamin use (%)	484 (25.7)	198 (21.6)	286 (29.7)	< 0.001^b^
Type 2 diabetes (%)	166 (8.6)	106 (11.2)	60 (6.1)	< 0.001^b^
Cardiovascular disease (%)	205 (10.3)	137 (14.1)	68 (6.7)	< 0.001^b^
Hypertension^d^ (%)	813 (45.1)	405 (45.3)	408 (44.9)	0.892^b^
BMI, kg/m^2^ (mean ± SD)	28.6 ± 4.7	29.1 ± 4.1	28.0 ± 5.1	< 0.001^a^
Energy intake, kcal [median (IQR)]	1907.3 (1482.0–2438.4)	1924.9 (1498.9–2476.7)	1874.9 (1466.6–2408.3)	0.114^c^
Dietary scores				
PDI score (mean ± SD)	51.1 ± 6.0	50.6 ± 6.1	51.6 ± 5.8	< 0.001^a^
hPDI score (mean ± SD)	52.8 ± 7.1	51.7 ± 7.1	53.9 ± 6.9	< 0.001^a^
uPDI score (mean ± SD)	52.0 ± 7.2	53.3 ± 6.9	50.8 ± 7.2	< 0.001^a^
Inflammatory biomarkers				
C3, mg/dL [median (IQR)]	134.11 (120.49–149.44)	132.77 (120.31–146.87)	135.52 (120.68–152.50)	0.003^c^
CRP, mg/L [median (IQR)]	1.35 (0.98–2.30)	1.32 (0.96–2.13)	1.38 (0.99–2.46)	0.041^c^
IL-6, pg/mL [median (IQR)]	1.78 (1.19–2.91)	1.92 (1.27–3.07)	1.68 (1.13–2.71)	< 0.001^c^
TNF-α, pg/mL [median (IQR)]	5.97 (4.89–7.29)	6.09 (5.05–7.49)	5.90 (4.76–7.15)	< 0.001^c^
Adiponectin, µg/mL [median (IQR)]	4.75 (2.92–7.55)	3.30 (2.21–4.92)	6.66 (4.45–9.62)	< 0.001^c^
Leptin, ng/mL [median (IQR)]	1.95 (1.09–3.14)	1.60 (0.83–2.60)	2.24 (1.27–4.25)	< 0.001^c^
LAR [median (IQR)]	0.41 (0.18–0.85)	0.47 (0.21–0.87)	0.36 (0.15–0.80)	< 0.001^c^
Resistin, ng/mL [median (IQR)]	5.05 (3.92–6.68)	4.88 (3.82–6.50)	5.22 (3.99–6.96)	0.003^c^
PAI-1, ng/mL [median (IQR)]	25.97 (19.16–33.98)	27.65 (20.15–35.88)	24.50 (17.78–32.03)	< 0.001^c^
WBC, 10^9^/L [median (IQR)]	5.70 (4.80–6.80)	5.90 (5.10–7.10)	5.50 (4.60–6.50)	< 0.001^c^
Neutrophils, 10^9^/L [median (IQR)]	3.12 (2.52–3.93)	3.28 (2.66–4.15)	2.98 (2.38–3.76)	< 0.001^c^
Lymphocytes, 10^9^/L [median (IQR)]	1.74 (1.42–2.14)	1.73 (1.41–2.14)	1.76 (1.44–2.15)	0.511^c^
NLR [median (IQR)]	1.78 (1.40–2.29)	1.86 (1.48–2.39)	1.67 (1.32–2.19)	< 0.001^c^
Monocytes, 10^9^/L [median (IQR)]	0.50 (0.40–0.62)	0.54 (0.44–0.68)	0.45 (0.37–0.56)	< 0.001^c^
Eosinophils, 10^9^/L [median (IQR)]	0.17 (0.11–0.26)	0.19 (0.12–0.28)	0.16 (0.10–0.24)	< 0.001^c^
Basophils, 10^9^/L [median (IQR)]	0.03 (0.02–0.04)	0.03 (0.02–0.04)	0.03 (0.02–0.04)	0.743^c^
GlycA, µmol/L [median (IQR)]	402.30 (367.40–445.70)	386.65 (352.98–424.03)	416.80 (380.60–460.10)	< 0.001^c^

Numbers and percentages may vary as some variables have missing values; Valid percentages are used where there is missing data

*BMI* body mass index, *C3* complement component 3, *CRP* C-reactive protein, *GlycA* glycoprotein A, *hPDI* healthful plant-based diet index, *IL-6* interleukin-6, *LAR* leptin-to-adiponectin ratio, *NLR* neutrophil-to-lymphocyte ratio, *PAI-1* plasminogen activator inhibitor, *PDI* plant-based diet index, *TNF-α* tumour necrosis factor alpha, *uPDI* unhealthful plant-based diet index, *WBC* white blood cell count

^a^Independent sample *t* test

^b^Pearson’s Chi-square test

^c^Mann–Whitney *U* Test

^d^Hypertension or using anti-hypertensive medication

Characteristics and inflammatory biomarker profiles of the study population examined according to PDI, hPDI and uPDI quintiles are presented in Tables 2 and 3. For the overall PDI, subjects with a more PBD (quintile 5) were less likely to be male, older, have a lower level of education, be heavy alcohol drinkers, live with type 2 diabetes, have a higher BMI, lower energy intake and have higher concentrations of CRP, IL-6, leptin, LAR, WBCs, neutrophils and monocytes and lower concentrations of adiponectin (all *P* < 0.05). For the hPDI, subjects with a more healthy PBD (quintile 5) were also less likely to be male, younger, current smokers, to not use multivitamins, have lower levels of physical activity, have a higher BMI and energy intake and have higher concentrations of C3, CRP, IL-6, TNF-α, LAR, PAI-1, WBCs, neutrophils, lymphocytes, monocytes and eosinophils and lower concentrations of adiponectin (all *P* < 0.05). For the uPDI, subjects with a more unhealthy PBD (quintile 5) were more likely to be male, to have achieved a lower level of education, be current smokers, have lower levels of physical activity, to not use multivitamins, have a lower energy intake and have higher concentrations of C3, CRP, IL-6, TNF-α, resistin, WBC, neutrophils, lymphocytes, NLR, monocytes and eosinophils and lower concentrations of adiponectin (all *P* < 0.05).

**Table 2:** Characteristics of the study population according to the PDI, hPDI and uPDI dietary score quintiles

Variable	PDI (*n* = 1986)			hPDI (*n* = 1986)			uPDI (*n* = 1986)		
	Q1 (*n* = 439)	Q5 (*n* = 365)	*P*	Q1 (*n* = 472)	Q5 (*n* = 364)	*P*	Q1 (*n* = 440)	Q5 (*n* = 381)	*P*
General									
Age, years [median (IQR)]	60.3 (55.6, 64.8)	58.1 (54.5, 63.4)	0.002^c^	57.8 (54.0, 63.2)	60.7 (55.9, 65.3)	< 0.001^b^	59.0 (54.6, 63.7)	60.2 (55.2, 64.7)	0.143^c^
Males (%)	248 (56.5)	164 (44.9)	0.001^b^	289 (61.2)	146 (40.1)	< 0.001^b^	160 (36.4)	229 (60.1)	< 0.001^b^
Primary education (%)	146 (35.3)	80 (23.2)	< 0.001^b^	117 (26.2)	89 (26.1)	0.966^b^	79 (19.3)	137 (38.0)	< 0.001^b^
Secondary education (%)	187 (45.2)	178 (51.6)	0.078^b^	224 (50.2)	152 (44.6)	0.116^b^	209 (51.0)	169 (46.8)	0.249^b^
Tertiary education (%)	81 (19.6)	87 (25.2)	0.062^b^	105 (23.5)	100 (29.3)	0.067^b^	122 (29.8)	55 (15.2)	< 0.001^b^
Current smoker (%)	71 (16.5)	47 (13.1)	0.185^b^	80 (17.4)	40 (11.2)	0.013^b^	40 (9.3)	62 (16.9)	0.001^b^
Non-drinker (%)	45 (14.9)	44 (18.8)	0.222^b^	44 (13.8)	40 (18.0)	0.177^b^	59 (19.0)	34 (14.5)	0.166^b^
Moderate drinker (%)	182 (60.1)	159 (67.9)	0.06^b^	220 (68.8)	152 (68.5)	0.945^b^	216 (69.5)	160 (68.1)	0.732^b^
Heavy drinker (%)	76 (25.1)	31 (13.2)	< 0.001^b^	56 (17.5)	30 (13.5)	0.212^b^	36 (11.6)	41 (17.4)	0.051^b^
Low-level physical activity (%)	203 (50.2)	162 (46.2)	0.262^b^	229 (52.2)	145 (42.2)	0.005^b^	179 (42.4)	205 (58.2)	< 0.001^b^
Moderate-level physical activity (%)	114 (28.2)	121 (34.5)	0.064^b^	110 (25.1)	126 (36.6)	< 0.001^b^	141 (33.4)	84 (23.9)	0.004^b^
High-level physical activity (%)	87 (21.5)	68 (19.4)	0.463^b^	100 (22.8)	73 (21.2)	0.602^b^	102 (24.2)	63 (17.9)	0.034^b^
Anti-inflammatory medication use (%)	88 (20.0)	63 (17.3)	0.314^b^	83 (17.6)	69 (19.0)	0.61^b^	87 (19.8)	73 (19.2)	0.825^b^
Multivitamin use (%)	106 (25.5)	99 (28.8)	0.318^b^	93 (20.6)	98 (28.5)	0.01^b^	121 (28.9)	77 (21.5)	0.019^b^
Type 2 diabetes (%)	49 (11.5)	22 (6.2)	0.01^b^	47 (10.2)	29 (8.2)	0.328^b^	38 (8.9)	36 (9.5)	0.735^b^
Cardiovascular disease (%)	51 (11.6)	31 (8.5)	0.145^b^	51 (10.8)	38 (10.4)	0.865^b^	45 (10.2)	42 (11.0)	0.712^b^
Hypertension^d^ (%)	171 (42.4)	148 (45.0)	0.489^b^	185 (42.5)	143 (45.0)	0.505^b^	180 (45.7)	161 (45.0)	0.845^b^
BMI, kg/m^2^ (mean ± SD)	29.3 ± 4.7	27.9 ± 4.3	< 0.001^a^	29.1 ± 4.7	27.8 ± 4.4	< 0.001^a^	28.1 ± 4.7	28.7 ± 4.7	0.11^a^
Energy intake, kcal [median (IQR)]	1611.0 (1243.5–2032.4)	2299.9 (1854.7–2864.8)	< 0.001^c^	2360.5 (1861.0–2947.2)	1594.0 (1239.9–1959.6)	< 0.001^c^	2281.0 (1871.3–2878.0)	1515.0 (1191.1–1938.7)	< 0.001^c^
Dietary scores									
Dietary scores [median (IQR)]	41 (38–43)	59 (57–61)	< 0.001^c^	42 (39–44)	61 (59–64)	< 0.001^c^	41 (38–43)	61 (59–64)	< 0.001^c^

Numbers and percentages may vary as some variables have missing values; Valid percentages are used where there is missing data

*BMI* body mass index, *hPDI* healthful plant-based diet index, *PDI* plant-based diet index, *uPDI* unhealthful plant-based diet index

^a^Independent sample *t* test

^b^Pearson’s Chi square test

^c^Mann–Whitney *U* test

^d^Hypertension or using anti-hypertensive medication

**Table 3:** Inflammatory biomarkers of the study population according to the PDI, hPDI and uPDI dietary score quintiles

Variable	PDI (*n* = 1986)			hPDI (*n* = 1986)			uPDI (*n* = 1986)		
	Q1 (*n* = 439)	Q5 (*n* = 365)	*P*	Q1 (*n* = 472)	Q5 (*n* = 364)	*P*	Q1 (*n* = 440)	Q5 (*n* = 381)	*P*
Inflammatory biomarkers [median (IQR)]									
C3, mg/dL	134.92 (123.12–152.11)	132.37 (120.19–146.99)	0.063^a^	135.81 (122.73–151.94)	130.95 (117.52–146.89)	0.002^a^	132.18 (118.01–144.65)	136.77 (123.90–152.31)	< 0.001^a^
CRP, mg/L	1.42 (0.97–2.40)	1.27 (0.91–2.05)	0.011^a^	1.40 (1.01–2.29)	1.29 (0.92–2.14)	0.031^a^	1.24 (0.93–1.92)	1.43 (1.02–2.57)	< 0.001^a^
IL-6, pg/mL	1.98 (1.27–3.09)	1.66 (1.19–2.42)	0.003^a^	1.94 (1.24–3.05)	1.65 (1.10–2.81)	0.014^a^	1.70 (1.14–2.64)	1.99 (1.33–3.18)	< 0.001^a^
TNF-α, pg/mL	6.14 (4.96–7.44)	5.89 (4.85–7.16)	0.067^a^	6.25 (5.07–7.63)	5.74 (4.74–6.99)	< 0.001^a^	5.77 (4.74–7.01)	6.24 (4.86–7.63)	0.003^a^
Adiponectin, µg/mL	4.37 (2.75–6.83)	4.89 (3.08–7.41)	0.029^a^	4.00 (2.59–6.30)	5.37 (3.31–8.31)	< 0.001^a^	5.33 (3.07–8.51)	4.15 (2.73–6.89)	< 0.001^a^
Leptin, ng/mL	2.05 (1.17–3.34)	1.89 (0.87–2.83)	0.026^a^	1.88 (1.20–2.91)	1.85 (1.00–2.95)	0.399^a^	1.86 (1.06–3.18)	1.86 (1.00–3.00)	0.569^a^
LAR	0.50 (0.23–0.94)	0.37 (0.16–0.75)	< 0.001^a^	0.50 (0.24–0.94)	0.33 (0.15–0.74)	< 0.001^a^	0.36 (0.15–0.80)	0.43 (0.19–0.82)	0.136^a^
Resistin, ng/mL	4.83 (3.83–6.48)	5.08 (3.92–6.75)	0.241^a^	5.26 (3.95–6.75)	4.96 (3.91–6.78)	0.49^a^	4.78 (3.79–6.50)	5.29 (3.94–6.86)	0.017^a^
PAI-1, ng/mL	25.73 (18.92–33.40)	25.36 (18.72–33.51)	0.951^a^	26.93 (19.86–35.20)	25.22 (18.40–32.39)	0.014^a^	25.09 (18.05–33.46)	26.31 (19.26–35.51)	0.078^a^
GlycA, µmol/L	403.30 (370.45–447.65)	397.60 (362.80–442.50)	0.219^a^	395.10 (363.73–438.38)	402.00 (366.10–444.60)	0.424^a^	399.40 (368.10–438.63)	401.25 (366.40–450.13)	0.526^a^
WBC, 10^9^/L	5.90 (5.00–6.90)	5.60 (4.80–6.70)	0.027^a^	5.90 (5.10–6.95)	5.50 (4.60–6.50)	< 0.001^a^	5.40 (4.40–6.40)	6.00 (5.10–7.10)	< 0.001^a^
Neutrophils, 10^9^/L	3.23 (2.60–4.08)	3.07 (2.47–3.83)	0.017^a^	3.30 (2.64–4.03)	2.93 (2.37–3.78)	< 0.001^a^	2.90 (2.24–3.66)	3.33 (2.71–4.13)	< 0.001^a^
Lymphocytes, 10^9^/L	1.78 (1.44–2.18)	1.76 (1.46–2.16)	0.978^a^	1.79 (1.46–2.20)	1.70 (1.43–2.09)	0.026^a^	1.72 (1.38–2.13)	1.79 (1.44–2.20)	0.019^a^
NLR	1.78 (1.40–2.39)	1.81 (1.37–2.19)	0.182^a^	1.78 (1.44–2.35)	1.79 (1.37–2.27)	0.241^a^	1.65 (1.31–2.21)	1.82 (1.47–2.30)	0.002^a^
Monocytes, 10^9^/L	0.52 (0.41–0.64)	0.48 (0.40–0.60)	0.015^a^	0.51 (0.42–0.64)	0.46 (0.38–0.59)	< 0.001^a^	0.46 (0.38–0.59)	0.51 (0.42–0.65)	< 0.001^a^
Eosinophils, 10^9^/L	0.18 (0.12–0.27)	0.17 (0.11–0.27)	0.197^a^	0.19 (0.13–0.27)	0.16 (0.11–0.24)	0.002^a^	0.16 (0.11–0.24)	0.19 (0.13–0.28)	< 0.001^a^
Basophils, 10^9^/L	0.03 (0.02–0.04)	0.03 (0.02–0.04)	0.999 ^a^	0.03 (0.02–0.04)	0.03 (0.02–0.04)	0.2^a^	0.03 (0.02–0.04)	0.03 (0.02–0.04)	0.299^a^

Numbers and percentages may vary as some variables have missing values

*C3* complement component 3, *CRP* C-reactive protein, *GlycA* glycoprotein A, *hPDI* healthful plant-based diet index, *IL-6* interleukin-6, *LAR* leptin-to-adiponectin ratio, *NLR* neutrophil-to-lymphocyte ratio, *PAI-1* plasminogen activator inhibitor, *PDI* plant-based diet index, *TNF-α* tumour necrosis factor alpha, *uPDI* unhealthful plant-based diet index, *WBC* white blood cell count

^a^Mann–Whitney *U* test

### Linear regression

Linear regression models examining the relationships between PDI, hPDI and uPDI scores and inflammatory profiles are presented in Tables 4 and 5. Sex- and age-adjusted associations observed between the overall PDI and lower concentrations of inflammatory biomarkers (C3, CRP, IL-6, leptin and LAR) did not persist in the fully adjusted model. Sex- and age-adjusted associations observed between the hPDI and lower concentrations of inflammatory biomarkers (C3, CRP, IL-6, TNF-α, leptin, PAI-1, WBCs, neutrophils, lymphocytes, monocytes, eosinophils and the LAR) persisted for C3, TNF-α, WBCs, neutrophils and monocytes. The association between the hPDI score and higher concentrations of adiponectin from the sex- and age-adjusted model was attenuated in the fully adjusted model. Sex- and age-adjusted associations observed between the uPDI and higher concentrations of inflammatory biomarkers (C3, CRP, IL-6, TNF-α, resistin, glycA, WBCs, neutrophils, lymphocytes, monocytes and eosinophils) persisted for C3 and TNF-α. After accounting for multiple testing, none of the observed relationships between the hPDI or uPDI scores and inflammatory biomarkers or WBC profiles persisted.

**Table 4:** Linear regression analysis of the associations between the PDI, hPDI and uPDI dietary scores and inflammatory biomarkers

Biomarker	PDI		hPDI		uPDI	
	*β* (95% CI)	*P*	*β* (95% CI)	*P*	*β* (95% CI)	*P*
C3						
Model 1	− 0.180 (− 0.360, 0.001)	0.051	− 0.252 (− 0.404, − 0.101)	0.001**	0.221 (0.071, 0.372)	0.004**
Model 2	− 0.197 (− 0.378, − 0.016)	0.033*	− 0.312 (− 0.467, − 0.158)	< 0.001***	0.265 (0.113, 0.417)	< 0.001***
Model 3	− 0.059 (− 0.285, 0.167)	0.609	− 0.201 (− 0.399, − 0.002)	0.048*	0.297 (0.098, 0.496)	0.003**
Model 4	− 0.147 (− 0.376, 0.083)	0.21	− 0.302 (− 0.496, − 0.107)	0.002**	0.237 (0.042, 0.432)	0.017*
CRP^a^						
Model 1	− 0.002 (− 0.005, − 0.000)	0.034*	− 0.002 (− 0.004, − 0.000)	0.019*	0.003 (0.001, 0.005)	0.005**
Model 2	− 0.002 (− 0.005, − 0.000)	0.047*	− 0.003 (− 0.005, − 0.001)	< 0.001***	0.003 (0.001, 0.005)	0.002**
Model 3	0.000 (− 0.003, 0.003)	0.98	− 0.002 (− 0.004, 0.001)	0.147	0.002 (− 0.000, 0.005)	0.105
Model 4	− 0.001 (− 0.004, 0.002)	0.425	− 0.003 (− 0.005, − 0.000)	0.022*	0.002 (− 0.001, 0.004)	0.145
IL-6^a^						
Model 1	− 0.004 (− 0.006, − 0.001)	0.002**	− 0.003 (− 0.005, − 0.001)	0.011*	0.003 (0.001, 0.005)	< 0.001***
Model 2	− 0.003 (− 0.005, − 0.000)	0.031*	− 0.003 (− 0.005, − 0.001)	0.002**	0.002 (0.000, 0.004)	0.024*
Model 3	− 0.000 (− 0.003, 0.003)	0.9	− 0.002 (− 0.004, 0.001)	0.205	0.001 (− 0.001, 0.004)	0.36
Model 4	− 0.001 (− 0.004, 0.002)	0.5	− 0.002 (− 0.005, 0.000)	0.059	0.001 (− 0.001, 0.004)	0.409
TNF-α^a^						
Model 1	− 0.001 (− 0.002, 0.000)	0.134	− 0.002 (− 0.003, − 0.001)	< 0.001***	0.002 (0.001, 0.003)	< 0.001***
Model 2	− 0.000 (− 0.002, 0.001)	0.433	− 0.002 (− 0.003, − 0.001)	< 0.001***	0.001 (0.000, 0.002)	0.011*
Model 3	− 0.000 (− 0.002, 0.001)	0.656	− 0.002 (− 0.003, − 0.001)	0.002**	0.002 (0.000, 0.003)	0.014*
Model 4	0.000 (− 0.002, 0.001)	0.808	− 0.002 (− 0.004, − 0.001)	< 0.001***	0.001 (− 0.000, 0.002)	0.097
Adiponectin^a^						
Model 1	0.002 (0.000, 0.005)	0.043*	0.006 (0.004, 0.008)	< 0.001***	− 0.004 (− 0.006, − 0.002)	< 0.001***
Model 2	0.001 (− 0.001, 0.003)	0.456	0.003 (0.001, 0.004)	0.003**	0.000 (− 0.002, 0.001)	0.727
Model 3	− 0.000 (− 0.003, 0.002)	0.91	0.002 (− 0.001, 0.004)	0.174	− 0.001 (− 0.003, 0.001)	0.499
Model 4	0.000 (− 0.002, 0.003)	0.722	0.002 (0.000, 0.005)	0.028*	0.000 (− 0.003, 0.002)	0.67
Leptin^a^						
Model 1	− 0.002 (− 0.005, 0.001)	0.146	− 0.001 (− 0.004, 0.001)	0.338	− 0.002 (− 0.004, 0.001)	0.195
Model 2	− 0.003 (− 0.006, − 0.001)	0.019*	− 0.004 (− 0.006, − 0.001)	0.003**	0.001 (− 0.002, 0.003)	0.539
Model 3	− 0.000 (− 0.004, 0.003)	0.826	− 0.001 (− 0.004, 0.002)	0.381	0.000 (− 0.003, 0.003)	0.825
Model 4	− 0.003 (− 0.007, 0.000)	0.09	− 0.004 (− 0.007, − 0.001)	0.017*	0.001 (− 0.003, 0.004)	0.715
LAR^a^						
Model 1	− 0.005 (− 0.008, − 0.001)	0.017*	− 0.007 (− 0.011, − 0.004)	< 0.001***	0.002 (− 0.001, 0.005)	0.194
Model 2	− 0.004 (− 0.008, − 0.000)	0.028*	− 0.006 (− 0.009, − 0.003)	< 0.001***	0.001 (− 0.002, 0.004)	0.511
Model 3	− 0.000 (− 0.004, 0.004)	0.935	− 0.003 (− 0.006, 0.001)	0.122	0.001 (− 0.003, 0.005)	0.548
Model 4	− 0.004 (− 0.008, 0.001)	0.137	− 0.006 (− 0.010, − 0.002)	0.002**	0.001 (− 0.003, 0.005)	0.612
Resistin^a^						
Model 1	0.001 (− 0.001, 0.002)	0.321	− 0.001 (− 0.002, 0.001)	0.381	0.001 (0.000, 0.002)	0.031*
Model 2	0.001 (− 0.001, 0.002)	0.360	− 0.001 (− 0.002, 0.000)	0.104	0.002 (0.000, 0.003)	0.009**
Model 3	0.001 (− 0.001, 0.003)	0.172	− 0.001 (− 0.002, 0.001)	0.407	0.002 (− 0.000, 0.003)	0.059
Model 4	0.001 (− 0.001, 0.003)	0.284	− 0.001 (− 0.002, 0.001)	0.277	0.001 (− 0.000, 0.003)	0.088
PAI− 1						
Model 1	− 0.026 (− 0.120, 0.067)	0.58	− 0.117 (− 0.196, − 0.038)	0.004**	0.104 (0.026, 0.182)	0.009**
Model 2	− 0.005 (− 0.098, 0.089)	0.923	− 0.084 (− 0.164, − 0.004)	0.04*	0.066 (− 0.013, 0.144)	0.103
Model 3	0.059 (− 0.066, 0.185)	0.352	− 0.058 (− 0.168, 0.052)	0.303	0.047 (− 0.064, 0.157)	0.406
Model 4	0.033 (− 0.086, 0.153)	0.585	− 0.074 (− 0.176, 0.028)	0.154	0.038 (− 0.064, 0.140)	0.461
GlycA						
Model 1	− 0.265 (− 0.744, 0.214)	0.278	0.079 (− 0.325, 0.482)	0.702	0.076 (− 0.323, 0.475)	0.708
Model 2	− 0.401 (− 0.869, 0.067)	0.093	− 0.337 (− 0.737, 0.063)	0.099	0.408 (0.014, 0.802)	0.042*
Model 3	− 0.018 (− 0.634, 0.598)	0.954	− 0.163 (− 0.705, 0.379)	0.554	0.133 (− 0.410, 0.677)	0.63
Model 4	− 0.160 (− 0.749, 0.428)	0.593	− 0.198 (− 0.700, 0.303)	0.437	0.166 (− 0.335, 0.666)	0.516

Model 1: crude; Model 2: adjusted for sex and age; Model 3: adjusted for sex, age, education, smoking status, alcohol consumption, physical activity, anti-inflammatory medication use, multivitamin use, type 2 diabetes status, cardiovascular disease status, hypertension or anti-hypertensive medication use, BMI and energy intake; Model 4: model 3 excluding BMI and energy intake

*C3* complement component 3, *CRP* C-reactive protein, *GlycA* glycoprotein A, *hPDI* healthful plant-based diet index, *IL-6* interleukin-6, *LAR* leptin-to-adiponectin ratio, *PAI-1* plasminogen activator inhibitor 1, *PDI* plant-based diet index, *TNF-α* tumour necrosis factor alpha, *uPDI* unhealthful plant-based diet index

*P < 0.05, **P < 0.01, ***P < 0.001

^a^Log-transformed

**Table 5:** Linear regression analysis of the associations between the PDI, hPDI and uPDI dietary scores and white blood cell profiles

Biomarker	PDI		hPDI		uPDI	
	*β* (95% CI)	*P*	*β* (95% CI)	*P*	*β* (95% CI)	*P*
WBC^a^						
Model 1	− 0.001 (− 0.002, − 0.000)	0.027*	− 0.002 (− 0.003, − 0.001)	< 0.001***	0.002 (0.001, 0.003)	< 0.001***
Model 2	− 0.001 (− 0.002, 0.000)	0.102	− 0.002 (− 0.002, − 0.001)	< 0.001***	0.002 (0.001, 0.002)	< 0.001***
Model 3	0.000 (− 0.001, 0.001)	0.481	− 0.001 (− 0.002, − 0.000)	0.014*	0.001 (− 0.000, 0.002)	0.063
Model 4	0.000 (− 0.001, 0.001)	0.838	− 0.001 (− 0.002, − 0.000)	0.021*	0.001 (0.000, 0.002)	0.027*
Neutrophils^a^						
Model 1	− 0.001 (− 0.002, − 0.000)	0.016*	− 0.002 (− 0.003, − 0.001)	< 0.001***	0.003 (0.002, 0.004)	< 0.001***
Model 2	− 0.001 (− 0.002, 0.000)	0.075	− 0.002 (− 0.003, − 0.001)	< 0.001***	0.002 (0.001, 0.003)	< 0.001***
Model 3	0.000 (− 0.001, 0.002)	0.599	− 0.001 (− 0.003, − 0.000)	0.025*	0.001 (− 0.000, 0.002)	0.065
Model 4	0.000 (− 0.002, 0.001)	0.688	− 0.001 (− 0.002, − 0.000)	0.036*	0.001 (0.000, 0.002)	0.026*
Lymphocytes^a^						
Model 1	0.000 (− 0.001, 0.001)	0.442	− 0.001 (− 0.002, − 0.000)	0.005**	0.001 (0.000, 0.002)	0.04*
Model 2	− 0.001 (− 0.002, 0.001)	0.314	− 0.001 (− 0.002, − 0.000)	0.013*	0.001 (0.000, 0.002)	0.024*
Model 3	0.000 (− 0.001, 0.002)	0.857	− 0.001 (− 0.002, 0.001)	0.289	0.001 (− 0.001, 0.002)	0.388
Model 4	0.000 (− 0.001, 0.001)	0.826	− 0.001 (− 0.002, 0.000)	0.188	0.001 (− 0.001, 0.002)	0.381
NLR^a^						
Model 1	− 0.001 (− 0.002, 0.000)	0.148	− 0.001 (− 0.002, 0.000)	0.138	0.002 (0.001, 0.003)	0.002**
Model 2	0.000 (− 0.002, 0.001)	0.488	− 0.001 (− 0.002, 0.000)	0.264	0.001 (− 0.000, 0.002)	0.058
Model 3	0.000 (− 0.001, 0.002)	0.78	− 0.001 (− 0.002, 0.001)	0.349	0.001 (− 0.001, 0.002)	0.432
Model 4	0.000 (− 0.002, 0.002)	0.883	0.000 (− 0.002, 0.001)	0.52	0.001 (− 0.001, 0.002)	0.267
Monocytes^a^						
Model 1	− 0.002 (− 0.003, − 0.001)	0.002 **	− 0.002 (− 0.003, − 0.001)	< 0.001***	0.002 (0.001, 0.003)	< 0.001***
Model 2	− 0.001 (− 0.002, 0.000)	0.066	− 0.001 (− 0.002, − 0.000)	0.003**	0.001 (0.000, 0.002)	0.002**
Model 3	0.000 (− 0.001, 0.002)	0.786	− 0.001 (− 0.002, − 0.000)	0.047*	0.000 (− 0.001, 0.002)	0.519
Model 4	0.000 (− 0.002, 0.001)	0.688	− 0.001 (− 0.002, 0.000)	0.11	0.001 (− 0.000, 0.002)	0.251
Eosinophils^a^						
Model 1	− 0.001 (− 0.003, 0.001)	0.55	− 0.002 (− 0.004, − 0.001)	0.006**	0.003 (0.002, 0.005)	< 0.001***
Model 2	− 0.000 (− 0.002, 0.002)	0.972	− 0.002 (− 0.004, − 0.000)	0.039*	0.002 (0.001, 0.004)	0.004**
Model 3	0.001 (− 0.001, 0.004)	0.361	− 0.002 (− 0.004, 0.000)	0.107	0.002 (− 0.001, 0.004)	0.171
Model 4	0.000 (− 0.002, 0.003)	0.763	− 0.001 (− 0.003, 0.001)	0.246	0.002 (− 0.000, 0.004)	0.073
Basophils						
Model 1	− 0.000 (− 0.000, 0.000)	0.412	− 0.000 (− 0.000, 0.000)	0.091	0.000 (− 0.000, 0.000)	0.224
Model 2	− 0.000 (− 0.000, 0.000)	0.400	− 0.000 (− 0.000, 0.000)	0.085	0.000 (− 0.000, 0.000)	0.209
Model 3	− 0.000 (− 0.000, 0.000)	0.659	− 0.000 (− 0.000, 0.000)	0.415	0.000 (− 0.000, 0.000)	0.738
Model 4	− 0.000 (− 0.000, 0.000)	0.684	− 0.000 (− 0.000, 0.000)	0.411	0.000 (− 0.000, 0.000)	0.794

Model 1: crude; Model 2: adjusted for sex and age; Model 3: adjusted for sex, age, education, smoking status, alcohol consumption, physical activity, anti-inflammatory medication use, multivitamin use, type 2 diabetes status, cardiovascular disease status, hypertension or anti-hypertensive medication use, BMI and energy intake; Model 4: model 3 excluding BMI and energy intake

*hPDI* healthful plant-based diet index, *NLR* neutrophil-to-lymphocyte ratio, *PDI* plant-based diet index, *uPDI* unhealthful plant-based diet index, *WBC* white blood cell count

*P < 0.05, **P < 0.01, ***P < 0.001

^a^Log-transformed

### Restricted and sensitivity analyses

Results of linear regression analysis excluding BMI and energy intake (model 4 presented in Tables 4 and 5), revealed associations between the hPDI and uPDI and inflammatory biomarkers which were similar to fully adjusted model 3 in the main analysis. For the hPDI, monocytes lost significance, while CRP, adiponectin, leptin and the LAR (which were significant in model 2 but attenuated in model 3) gained significance. For the uPDI, TNF-α lost significance, while WBCs and neutrophils (which were significant in model 2 but attenuated in model 3) gained significance.

It is noteworthy that in the analysis restricted to participants with plausible energy intakes according to sex-specific cut-offs, all the aforementioned results observed in the fully adjusted models remained (Supplemental Table 2). In a sensitivity analysis where boiled and mashed potatoes were scored in the vegetables food group, associations between the hPDI and uPDI and inflammatory biomarkers were virtually unchanged from the main analysis (Supplemental Tables 3 and 4).

## Discussion

To our knowledge this study is the most extensive examination of PDI score relationships with inflammatory biomarkers conducted to date. In regression analyses, higher hPDI scores, indicating a more healthful PBD, were associated with lower concentrations of C3, TNF-α, WBCs, neutrophils and monocytes. Higher uPDI scores, indicating a more unhealthful PBD, were associated with higher concentrations of C3 and TNF-α. These results were robust to a restricted analysis excluding participants with implausible energy intakes, and to a sensitivity analysis with boiled and mashed potatoes scored in the vegetables food group. This suggests that habitual consumption of healthy plant-based foods may positively influence inflammatory biomarkers, whereas the consumption of unhealthy plant-based and animal-based foods may be associated with an unfavourable inflammatory profile.

The importance of characterising the relationship between diet and inflammation through the assessment of dietary patterns has been previously highlighted [37]. However, few studies have examined associations between PDI scores and inflammatory markers [19, 20]. Baden et al. reported lower leptin and high-sensitivity CRP levels and higher adiponectin concentrations per 10-point increase in the hPDI and higher leptin levels per 10-unit increase in the uPDI in multivariable models [19]. In the current study, the hPDI was similarly associated with lower leptin, CRP and higher adiponectin concentrations, but only in crude and sex- and age-adjusted models. These differences may reflect diverging cohort characteristics including sex, energy intake, mean diet scores and differences in model adjustment. No studies thus far have assessed relationships between PDI scores and C3, TNF-α and WBC concentrations.

Elevated concentrations of C3, an acute-phase response protein, have been shown to be correlated with insulin, glucose, insulin resistance and associated with an increased risk of type 2 diabetes [38, 39]. TNF-α, a multifunctional circulating cytokine, has been linked with a higher risk of CVD and a lower risk of overall and certain cancers [40]. WBCs, a non-specific marker of inflammation, have been shown to be an independent risk factor for type 2 diabetes in subjects with increased adiposity. In addition, it has been observed that individuals with overweight and obesity with relatively low WBC concentrations have a significantly lower risk for diabetes than those with higher levels of leukocytes [41].

Our findings may partly explain results from previous studies examining PDI score associations with hard CVD endpoints; these have reported a lower risk of CVD events and mortality with higher PDI and hPDI scores and a higher risk with lower uPDI scores [10–13]. Baden et al. investigated adherence to the PDI, hPDI and uPDI using data from the NHS I and II and the HPFS, reporting a 7% and 9% reduced risk of CVD mortality for each 10-point increase in the PDI and hPDI and an 8% increase with each 10-point increase in the uPDI [11]. Another study reported that coronary heart disease (CHD) risk decreased by 7% and 12% with each 10-point increase in the PDI and hPDI and increased by 10% with each 10-point increase in uPDI [10]. Examination of hPDI associations with CVD outcomes using UK Biobank data revealed that a 10-point increase in the hPDI was linked with an 8%, 13% and 10% reduced risk of MI, stroke and CVD, respectively [12]. A separate, more recent analysis of the UK Biobank cohort reported similar findings [13]. Conversely, Weston et al. found no difference in CHD, MI, ischemic stroke or haemorrhagic stroke risk comparing adherence to the PDI scores [42].

The observed magnitude of difference in inflammatory biomarker concentrations comparing highest and lowest dietary index quintiles suggest potentially clinically important differences. The differences in WBC and neutrophil concentrations between the highest and lowest hPDI quintiles (7% and 12% for WBCs and neutrophils, respectively) are greater than those reported between non-CVD mortality and CVD mortality cases (6% and 7% for WBC and neutrophils, respectively) in the UK Biobank study [43]. Similarly, the difference in TNF-*α* concentrations between the highest and lowest hPDI quintiles (9%), is greater than those reported between non-cases and cases of a CHD event (8%) in the Procardis study [44]. The difference between highest and lowest hPDI quintiles of C3 (4%) exceeds that reported in those who did not experience a coronary event compared to those who did (3%) in a male Swedish cohort [45]. Even the difference in monocyte concentrations between the highest and lowest hPDI quintiles (10%) is greater than those reported between controls without CHD and individuals with CHD (3%) in the UK Biobank cohort [46].

Previous work by our group examined dietary score and inflammatory biomarker associations using a range of dietary scores reflecting dietary quality (Dietary Approach To Stop Hypertension [DASH] and Healthy Eating Index 2015 [HEI-2015]) and dietary inflammatory potential (DII and energy-adjusted DII™ [E-DII]) [22, 32, 47]. Phillips et al. reported that a more pro-inflammatory diet, indicated by higher E-DII scores was associated with higher concentrations of C3, CRP, IL-6, TNF-*α*, WBCs and lower concentrations of adiponectin [32]. Millar et al. reported inverse associations between the HEI-2015 and WBCs, neutrophil and CRP concentrations [22]. In a subsequent analysis of the DASH score, Mediterranean diet, DII and E-DII scores, Millar et al. also reported associations between higher dietary quality and an anti-inflammatory diet with lower concentrations of C3, TNF-*α*, WBCs, neutrophils, IL-6 and the NLR [47]. These finding are consistent with results from the current study.

Research on inflammatory markers and risk of CVD supports the view that a healthier PBD may reduce the risk of CVD outcomes, whereas a less healthy PBD increases risk. Of note, in the current study, both the PDI and hPDI scores were positively correlated with the DASH score (*ρ* = 0.231 and *ρ* = 0.588, respectively) and negatively correlated with the E-DII (*ρ* = − 0.288 and *ρ* = − 0.588, respectively), whereas the uPDI score was negatively correlated with the DASH score (*ρ* = − 0.435) and positively with the E-DII *(ρ* = 0.335) (all *P* < 0.001; data not shown). Considering our previous findings, these results suggest that higher hPDI scores reflect a more anti-inflammatory dietary pattern, whereas higher uPDI scores indicate a more pro-inflammatory dietary pattern. Interestingly, the overall PDI showed a poor ability to associate with inflammatory biomarkers, which suggests that a PBD per se is not associated with inflammation. Observing contingency tables, a minority of individuals were categorised in the same quintiles across scores, which may explain these findings (Supplemental Tables 5, 6 and 7). For example, only 27% of individuals in Q5 of the hPDI were also in Q5 of the PDI (Supplemental Table 5).

This study has several strengths including the relatively large number of middle- to older-aged study participants, the equal representation by sex (49% male), the use of a validated FFQ to collect dietary data and the examination of a wide range of inflammatory biomarkers. Notwithstanding these strengths some limitations should be noted. The cross-sectional design precludes causal inference and determination of temporality. Therefore, further research is needed to strengthen the evidence for these novel findings. In addition, the use of self-reported questionnaires is subject to potential inaccuracies such as recall and reporting bias. The diet scores in this study are dependent on within-cohort differences in dietary intakes, which may limit the extent to which associations could be captured. Indeed, our study population were observed to have homogeneous food group and nutrient intakes. Study populations with more diverse eating habits may have bigger contrasts in dietary intakes and this warrants further investigation. In addition, although we adjusted for a wide range of confounding variables, residual confounding may remain from imprecise measurements. Finally, the generalisability of our findings may be limited (particularly on an international scale) as the study data were collected from a single primary-care sample. However, Ireland’s population is generally ethnically homogeneous [48]. Previous research suggests that 98% of the Irish adult population are registered with a GP, making it possible to perform population-based epidemiological studies that are representative even in the absence of a universal patient registration system [49].

In conclusion, the results from this study suggest that a healthy PBD, characterised by the consumption of more healthy plant-based foods and less unhealthy plant-based foods and animal-based foods, is associated with a more favourable inflammatory biomarker profile in middle- to older-aged adults. Conversely, an unhealthy PBD, characterised by the consumption of more unhealthy plant-based foods and less healthy plant-based foods and animal-based foods is associated with an unfavourable, pro-inflammatory biomarker profile. These findings suggest reduced chronic low-grade inflammation as a potential biological mechanism underlying the protective role of PBDs in cardiometabolic health. Further, these findings underscore the importance of PBD quality, highlighting that not all PBDs are health-promoting, and that public health nutrition policy should underscore the importance of consuming a healthy PBD for optimal health outcomes. Future research examining the relationships between PBDs and other biomarkers of health is warranted with a view to informing public health nutrition policy and the promotion of healthy eating to improve dietary quality and overall health and well-being.

### Supplementary Information

Below is the link to the electronic supplementary material.

Supplementary file1 (DOCX 158 KB)

## Data Availability

Data described in the manuscript, code book, and analytic code are freely available to other researchers upon request.

## Abbreviations

BMI: Body mass index
C3: Complement component 3
CHD: Coronary heart disease
CRP: C-reactive protein
CVD: Cardiovascular disease
DASH: Dietary approach to stop hypertension
DII: Dietary inflammatory index
E-DII: Energy-Adjusted Dietary Inflammatory Index™
FFQ: Food frequency questionnaire
GHQ: General health questionnaire
GP: General practitioner
GlycA: Glycoprotein A
HEI-2015: Healthy Eating Index 2015
hPDI: Healthful plant-based diet index
HPFS: Health professionals follow-up study
IL-6: Interleukin-6
IPAQ: International Physical Activity Questionnaire
LAR: Leptin-to-adiponectin ratio
MI: Myocardial infarction
NCDs: Non-communicable diseases
NHS: Nurses’ Health Study
NLR: Neutrophil-to-lymphocyte ratio
PAI-1: Plasminogen activator inhibitor 1
PBD: Plant-based diet
PDI: Plant-based diet index
SSBs: Sugar sweetened beverages
TNF-α: Tumour necrosis factor alpha
uPDI: Unhealthful plant-based diet index
WBCs: White blood cells
