# Supplementary material for: Plant-based dietary indices and biomarkers of chronic low-grade inflammation: a cross-sectional analysis of adults in Ireland
Source: Eur J Nutr. 2023 Sep 2;62(8):3397–410. doi: 10.1007/s00394-023-03242-5 (PMC10611858; doi:10.1007/s00394-023-03242-5)
Supplement: Supplementary file 1 — Supplementary file1 (DOCX 158 KB) [file 394_2023_3242_MOESM1_ESM.docx]

**Title**: Plant-based dietary indices and biomarkers of chronic low-grade inflammation: A cross-sectional analysis of adults in Ireland.

**Journal:** European Journal of Nutrition

**Authors:**

Soraeya Kharaty, School of Public Health, Physiotherapy and Sports Science, University College Dublin, Dublin 4, Ireland Orcid ID: https://orcid.org/0000-0002-8021-3027

Janas M. Harrington, School of Public Health, University College Cork, Cork, Ireland Orcid ID: https://orcid.org/0000-0002-6238-7031

Seán R. Millar, School of Public Health, University College Cork, Cork, Ireland Orcid ID: https://orcid.org/0000-0003-4453-8446

Ivan J. Perry, School of Public Health, University College Cork, Cork, Ireland Orcid ID: https://orcid.org/0000-0002-4965-9792

Catherine M. Phillips, School of Public Health, Physiotherapy and Sports Science, University College Dublin, Dublin 4, Ireland Orcid ID: https://orcid.org/0000-0003-4916-4463

**Corresponding Author:** Catherine M. Phillips, School of Public Health, Physiotherapy and Sports Science, University College Dublin 4, Ireland. Email: catherine.phillips@ucd.ie


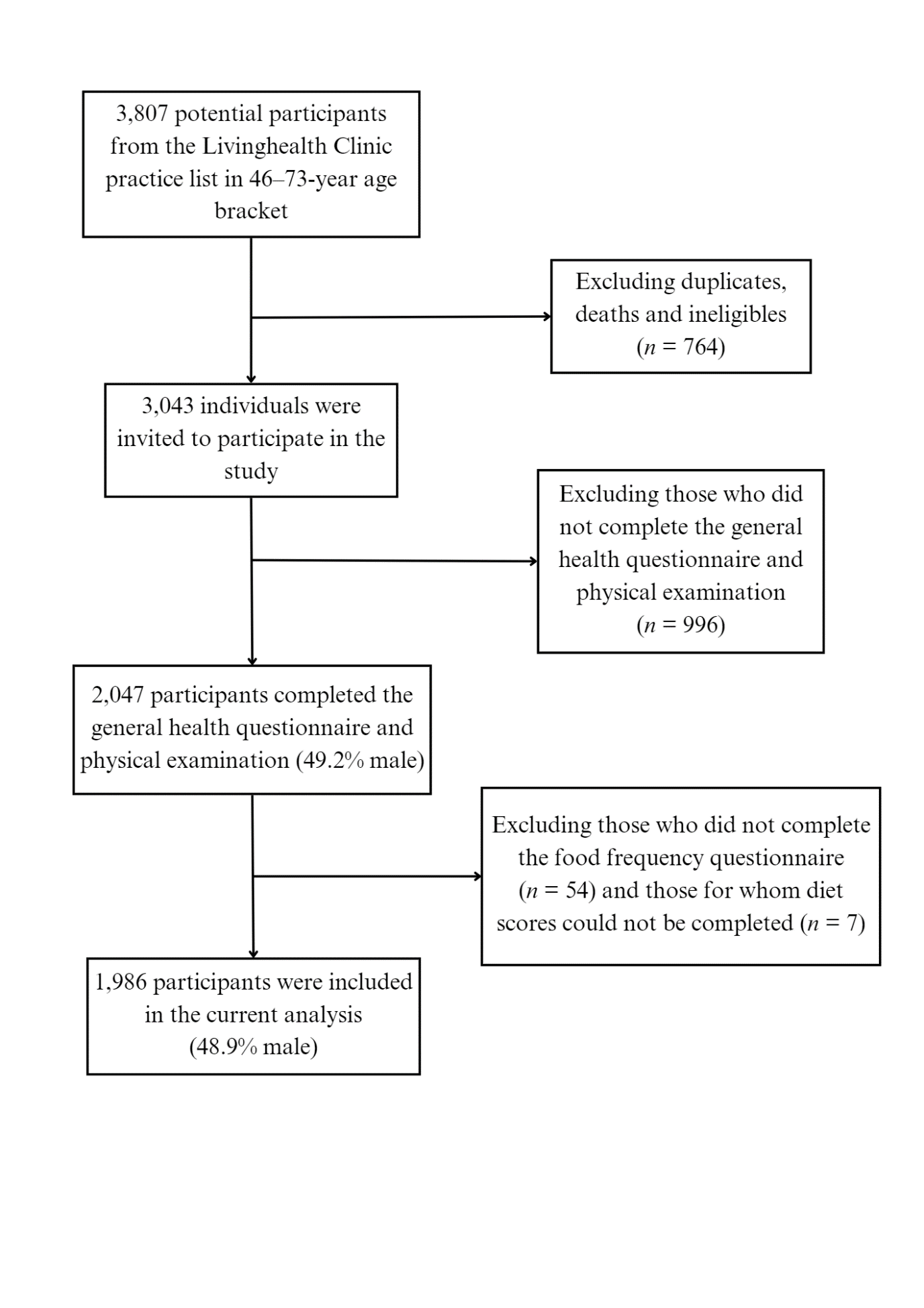


**Supplemental Figure 1: Flow chart outlining subject selection for the Mitchelstown Cohort**

**Supplemental Table 1: Spearman Correlation Coefficients Between the PDI, hPDI and uPDI Dietary Scores and Dietary and Nutrient Intakes**

| **Dietary and Nutrient Intakes** | PDI | | hPDI | | uPDI | |
| --- | --- | --- | --- | --- | --- | --- |
|  | *ρ* coefficient | *P* | *ρ*  coefficient | *P* | *ρ*  coefficient | *P* |
| **Macronutrients** |  |  |  |  |  |  |
| Fat (% kcal/day) | 0.013 | 0.58 | 0.005 | 0.837 | -0.007 | 0.762 |
| SFA (% fat kcal/day) | -0.030 | 0.188 | -0.011 | 0.638 | -0.019 | 0.404 |
| PUFA (% fat kcal/day) | 0.021 | 0.348 | 0.005 | 0.826 | 0.036 | 0.116 |
| MUFA (% fat kcal/day) | -0.002 | 0.938 | 0.004 | 0.866 | 0.001 | 0.953 |
| Carbohydrate (% kcal/day) | -0.044 | 0.055 | 0.009 | 0.699 | -0.020 | 0.385 |
| Protein (% kcal/day) | -0.004 | 0.874 | -0.052 | 0.023 ^*^ | 0.042 | 0.063 |
| Sugar (% kcal/day) | -0.007 | 0.753 | -0.001 | 0.956 | -0.012 | 0.587 |
| Fibre (% kcal/day) | 0.026 | 0.253 | 0.001 | 0.975 | -0.002 | 0.943 |
| Alcohol (% kcal/day) | 0.071 | 0.002 ^**^ | 0.008 | 0.729 | 0.014 | 0.528 |
| Cholesterol mg/d | -0.051 | 0.023 ^*^ | -0.511 | < 0.001 ^***^ | -0.418 | < 0.001 ^***^ |
| **Micronutrients** |  |  |  |  |  |  |
| Vitamin B12, μg/d | -0.046 | 0.041 ^*^ | -0.288 | < 0.001 ^***^ | -0.441 | < 0.001 ^***^ |
| Vitamin B6, mg/d | 0.324 | < 0.001 ^***^ | -0.231 | < 0.001 ^***^ | -0.435 | < 0.001 ^***^ |
| Folate, μg/d | 0.425 | < 0.001 ^***^ | -0.120 | < 0.001 ^***^ | -0.499 | < 0.001 ^***^ |
| Vitamin C, mg/d | 0.440 | < 0.001 ^***^ | 0.041 | 0.071 | -0.455 | < 0.001 ^***^ |
| Vitamin D, μg/d | 0.128 | < 0.001 ^***^ | -0.275 | < 0.001 ^***^ | -0.386 | < 0.001 ^***^ |
| Vitamin E, mg/d | 0.397 | < 0.001 ^***^ | 0.004 | 0.875 | -0.499 | < 0.001 ^***^ |
| Sodium, mg/d | 0.273 | < 0.001 ^***^ | -0.296 | < 0.001 ^***^ | -0.432 | < 0.001 ^***^ |
| Niacin, mg/d | 0.204 | < 0.001 ^***^ | -0.247 | < 0.001 ^***^ | -0.506 | < 0.001 ^***^ |
| Thiamine, mg/d | 0.375 | < 0.001 ^***^ | -0.242 | < 0.001 ^***^ | -0.418 | < 0.001 ^***^ |
| Riboflavin, mg/d | 0.218 | < 0.001 ^***^ | -0.261 | < 0.001 ^***^ | -0.528 | < 0.001 ^***^ |
| Iron, mg/d | 0.371 | < 0.001 ^***^ | -0.180 | < 0.001 ^***^ | -0.490 | < 0.001 ^***^ |
| Magnesium, mg/d | 0.383 | < 0.001 ^***^ | -0.077 | < 0.001 ^***^ | -0.603 | < 0.001 ^***^ |
| Zinc, mg/d | 0.198 | < 0.001 ^***^ | -0.286 | < 0.001 ^***^ | -0.474 | < 0.001 ^***^ |
| Selenium, μg/d | 0.142 | < 0.001 ^***^ | -0.398 | < 0.001 ^***^ | -0.367 | < 0.001 ^***^ |
| Retinol, μg/d | 0.050 | 0.026 ^*^ | -0.346 | < 0.001 ^***^ | -0.270 | < 0.001 ^***^ |
| Carotene, μg/d | 0.293 | < 0.001 ^***^ | 0.037 | 0.1 | -0.507 | < 0.001 ^***^ |
| **Food Groups (servings/day)** |  |  |  |  |  |  |
| Whole grains | 0.325 | < 0.001 ^***^ | 0.240 | < 0.001 ^***^ | -0.421 | < 0.001 ^***^ |
| Fruit | 0.346 | < 0.001 ^***^ | 0.232 | < 0.001 ^***^ | -0.504 | < 0.001 ^***^ |
| Vegetables | 0.321 | < 0.001 ^***^ | 0.162 | < 0.001 ^***^ | -0.585 | < 0.001 ^***^ |
| Nuts | 0.004 | 0.867 | -0.108 | < 0.001 ^***^ | -0.259 | < 0.001 ^***^ |
| Legumes | 0.318 | < 0.001 ^***^ | 0.025 | 0.26 | -0.336 | < 0.001 ^***^ |
| Vegetable Oils | 0.400 | < 0.001 ^***^ | 0.226 | < 0.001 ^***^ | -0.264 | < 0.001 ^***^ |
| Tea and Coffee | 0.320 | < 0.001 ^***^ | 0.104 | < 0.001 ^***^ | -0.300 | < 0.001 ^***^ |
| Fruit Juices | 0.357 | < 0.001 ^***^ | -0.295 | < 0.001 ^***^ | 0.082 | < 0.001 ^***^ |
| Refined Grains | 0.232 | < 0.001 ^***^ | -0.425 | < 0.001 ^***^ | 0.131 | < 0.001 ^***^ |
| Potatoes | 0.256 | < 0.001 ^***^ | -0.400 | < 0.001 ^***^ | 0.043 | 0.056 |
| Sugar Sweetened Beverages | 0.237 | < 0.001 ^***^ | -0.428 | < 0.001 ^***^ | 0.161 | < 0.001 ^***^ |
| Sweets and Desserts | 0.297 | < 0.001 ^***^ | -0.397 | < 0.001 ^***^ | 0.096 | < 0.001 ^***^ |
| Animal Fat | -0.204 | < 0.001 ^***^ | -0.459 | < 0.001 ^***^ | -0.019 | 0.399 |
| Dairy | -0.054 | 0.016 ^*^ | -0.278 | < 0.001 ^***^ | -0.384 | < 0.001 ^***^ |
| Eggs | -0.160 | < 0.001 ^***^ | -0.249 | < 0.001 ^***^ | -0.288 | < 0.001 ^***^ |
| Fish and Seafood | -0.082 | < 0.001^***^ | -0.136 | < 0.001 ^***^ | -0.434 | < 0.001 ^***^ |
| Meat | -0.065 | 0.004 ^**^ | -0.440 | < 0.001 ^***^ | -0.266 | < 0.001 ^***^ |
| Misc. animal-based foods | -0.020 | 0.377 | -0.329 | < 0.001 ^***^ | -0.417 | < 0.001 ^***^ |

**Abbreviations:** hPDI: healthful plant-based diet index; MUFA: monounsaturated fatty acids; PDI: plant-based diet index; PUFA: polyunsaturated fatty acids; SFA: saturated fatty acids; uPDI: unhealthful plant-based diet index; ^*^*P*<0.05, ^**^*P*<0.01, ^***^*P*<0.001.

**Supplemental Table 2: Sensitivity Analysis of the Associations Between the PDI, hPDI, and uPDI Dietary Scores and Inflammatory Biomarkers and White Blood Cell Profiles – Excluding Individuals with Implausible Energy Intakes using Sex-Specific Cut-Offs**

| **Biomarker** | PDI | | hPDI | | uPDI | |
| --- | --- | --- | --- | --- | --- | --- |
|  | *β* (95% CI) | *P* | *β* (95% CI) | *P* | *β* (95% CI) | *P* |
| C3 | -0.076 (-0.308, 0.157) | 0.522 | -0.208 (-0.412, -0.004) | 0.046 ^*^ | 0.296 (0.092, 0.500) | 0.004 ^**^ |
| CRP ^a^ | 0.000 (-0.003, 0.003) | 0.833 | -0.002 (-0.005, 0.000) | 0.076 | 0.002 (-0.000, 0.005) | 0.081 |
| IL-6 ^a^ | 0.000 (-0.003, 0.003) | 0.9 | -0.002 (-0.005, 0.000) | 0.105 | 0.001 (-0.001, 0.004) | 0.327 |
| TNF-*α* ^a^ | 0.000 (-0.002, 0.001) | 0.754 | -0.002 (-0.004, -0.001) | 0.002 ^**^ | 0.002 (0.000, 0.003) | 0.018 ^*^ |
| Adiponectin ^a^ | 0.000 (-0.002, 0.003) | 0.845 | 0.001 (-0.001, 0.004) | 0.258 | -0.001 (-0.003, 0.001) | 0.449 |
| Leptin ^a^ | -0.001 (-0.004, 0.003) | 0.721 | -0.001 (-0.004, 0.001) | 0.329 | 0.000 (-0.002, 0.003) | 0.769 |
| LAR ^a^ | -0.001 (-0.005, 0.003) | 0.704 | -0.003 (-0.007, 0.001) | 0.142 | 0.001 (-0.002, 0.005) | 0.482 |
| Resistin ^a^ | 0.001 (-0.001, 0.003) | 0.178 | -0.001 (-0.002, 0.001) | 0.394 | 0.001 (-0.000, 0.003) | 0.122 |
| PAI-1 | 0.059 (-0.070, 0.189) | 0.37 | -0.068 (-0.182, 0.046) | 0.242 | 0.058 (-0.056, 0.172) | 0.321 |
| Glycoprotein A | -0.004 (-0.636, 0.628) | 0.99 | -0.291 (-0.847, 0.265) | 0.304 | 0.032 (-0.525, 0.589) | 0.911 |
| WBC ^a^ | 0.000 (-0.001, 0.001) | 0.637 | -0.001 (-0.002, -0.000) | 0.008 ^**^ | 0.001 (-0.000, 0.002) | 0.114 |
| Neutrophils ^a^ | 0.000 (-0.001, 0.002) | 0.744 | -0.002 (-0.003, -0.000) | 0.022 ^*^ | 0.001 (-0.000, 0.002) | 0.084 |
| Lymphocytes ^a^ | 0.000 (-0.001, 0.001) | 0.975 | -0.001 (-0.002, 0.000) | 0.174 | 0.000 (-0.001, 0.002) | 0.674 |
| NLR ^a^ | 0.000 (-0.002, 0.002) | 0.808 | -0.001 (-0.002, 0.001) | 0.452 | 0.001 (-0.001, 0.002) | 0.292 |
| Monocytes ^a^ | 0.000 (-0.001, 0.001) | 0.868 | -0.001 (-0.002, -0.000) | 0.045 ^*^ | 0.000 (-0.001, 0.001) | 0.751 |
| Eosinophils ^a^ | 0.001 (-0.002, 0.004) | 0.459 | -0.002 (-0.005, 0.000) | 0.072 | 0.002 (-0.001, 0.004) | 0.15 |
| Basophils | -0.000 (-0.000, 0.000) | 0.615 | 0.000 (-0.000, 0.000) | 0.202 | 0.000 (-0.000, 0.000) | 0.922 |

**Abbreviations:** hPDI: healthful plant-based diet index; LAR: leptin-to-adiponectin ratio; NLR: neutrophil-to-lymphocyte ratio; PDI: plant-based diet index; uPDI: unhealthful plant-based diet index; WBC: white blood cell count; Implausible daily energy intakes defined as < 800 kcals and > 4,000 kcals for males and < 500 kcals and > 3,500 kcals for females. Coefficients adjusted for sex, age, education, smoking status, alcohol consumption, physical activity, anti-inflammatory medication use, multivitamin use, type 2 diabetes, cardiovascular disease, hypertension or anti-hypertension medication use, body mass index and energy intake; ^*^*P*<0.05, ^**^*P*<0.01, ^***^*P*< 0.001; ^a^Log-transformed.

**Supplemental Table 3: Sensitivity Analysis of Linear Regression Analysis of the Associations Between the PDI, hPDI, and uPDI Dietary Scores and Inflammatory Biomarkers – With Boiled and Mashed Potatoes Scored in the Vegetable Group**

**Abbreviations:** C3: complement component 3; CRP: C-reactive protein; hPDI: healthful plant-based diet index; IL-6: interleukin-6; LAR: leptin-to-adiponectin ratio; PAI-1: plasminogen activator inhibitor 1; TNF-α: tumour necrosis factor alpha; uPDI: unhealthful plant-based diet index; Coefficients adjusted for sex, age, education, smoking status, alcohol consumption, physical activity, anti-inflammatory medication use, multivitamin use, type 2 diabetes, cardiovascular disease, hypertension or anti-hypertension medication use, body mass index and energy intake; ^*^*P*<0.05, ^**^*P*<0.01, ^***^*P*<0.001; ^a^Log-transformed.

| **Biomarker** | hPDI | | uPDI | |
| --- | --- | --- | --- | --- |
|  | *β* (95% CI) | *P* | *β* (95% CI) | *P* |
| C3 | -0.215 (-0.407, -0.023) | 0.028 ^*^ | 0.340 (0.137, 0.543) | 0.001 ^**^ |
| CRP ^a^ | -0.002 (-0.004, 0.001) | 0.185 | 0.002 (-0.001, 0.005) | 0.114 |
| IL-6 ^a^ | -0.002 (-0.004, 0.001) | 0.249 | 0.001 (-0.002, 0.004) | 0.386 |
| TNF-α ^a^ | -0.002 (-0.003, -0.000) | 0.009 ^**^ | 0.001 (0.000, 0.003) | 0.031^*^ |
| Adiponectin ^a^ | 0.001 (-0.001, 0.004) | 0.233 | -0.001 (-0.003, 0.002) | 0.575 |
| Leptin ^a^ | -0.001 (-0.004, 0.002) | 0.402 | 0.000 (-0.003, 0.003) | 0.833 |
| LAR ^a^ | -0.003 (-0.006, 0.001) | 0.157 | 0.001 (-0.003, 0.005) | 0.602 |
| Resistin ^a^ | 0.000 (-0.002, 0.001) | 0.658 | 0.001 (-0.000, 0.003) | 0.121 |
| PAI-1 | -0.046 (-0.152, 0.061) | 0.399 | 0.040 (-0.073, 0.152) | 0.49 |
| Glycoprotein A | -0.084 (-0.607, 0.439) | 0.752 | 0.064 (-0.491, 0.619) | 0.821 |

**Supplemental Table 4: Sensitivity Analysis of Linear Regression Analysis of the Associations Between the PDI, hPDI, and uPDI Dietary Scores and White Blood Cell Profiles – With Boiled and Mashed Potatoes Scored in the Vegetable Group**

**Abbreviations:** hPDI: healthful plant-based diet index; NLR: neutrophil-to-lymphocyte ratio; uPDI: unhealthful plant-based diet index; WBC: white blood cell count; Coefficients adjusted for sex, age, education, smoking status, alcohol consumption, physical activity, anti-inflammatory medication use, multivitamin use, type 2 diabetes, cardiovascular disease, hypertension or anti-hypertension medication use, body mass index and energy intake; ^*^*P* < 0.05, ^**^*P* < 0.01, ^***^*P* < 0.001*; ^a^*Log-transformed.

| **Biomarker** | hPDI | | uPDI | |
| --- | --- | --- | --- | --- |
|  | *β* (95% CI) | *P* | *β* (95% CI) | *P* |
| WBC ^a^ | -0.001 (-0.002, -0.000) | 0.02 ^*^ | 0.001 (-0.000, 0.002) | 0.062 |
| Neutrophils ^a^ | -0.001 (-0.002, -0.000) | 0.05 ^*^ | 0.001 (-0.000, 0.002) | 0.092 |
| Lymphocytes ^a^ | -0.001 (-0.002, 0.000) | 0.165 | 0.001 (-0.000, 0.002) | 0.204 |
| NLR ^a^ | 0.000 (-0.002, 0.001) | 0.652 | 0.000 (-0.001, 0.002) | 0.741 |
| Monocytes ^a^ | -0.001 (-0.002, 0.000) | 0.07 | 0.000 (-0.001, 0.002) | 0.576 |
| Eosinophils ^a^ | -0.002 (-0.005, -0.000) | 0.05 ^*^ | 0.002 (-0.000, 0.005) | 0.067 |
| Basophils | -0.000 (-0.000, 0.000) | 0.381 | 0.000 (-0.000, 0.000) | 0.662 |

**Supplemental Table 5: Contingency Table Between Quintiles of PDI and hPDI**

| **PDI** | **hPDI** | |  | |  |
| --- | --- | --- | --- | --- | --- |
|  | Q1 | Q2 | Q3 | Q4 | Q5 |
| Q1 (*n* = 439) | 144 (32.8) | 98 (22.3) | 102 (23.2) | 61 (13.9) | 34 (7.7) |
| Q2 (*n* = 481) | 121 (25.2) | 107 (22.2) | 99 (20.6) | 94 (19.5) | 60 (12.5) |
| Q3 (*n* = 275) | 57 (20.7) | 52 (18.9) | 59 (21.5) | 44 (16.0) | 63 (22.9) |
| Q4 (*n* = 426) | 104 (24.4) | 67 (15.7) | 71 (16.7) | 76 (17.8) | 108 (25.4) |
| Q5 (*n* = 365) | 46 (12.6) | 64 (17.5) | 82 (22.5) | 74 (20.3) | 99 (27.1) |

**Abbreviations:** hPDI: healthful plant-based diet index; PDI: plant-based diet index; Counts (%) are presented within each quintile.

**Supplemental Table 6: Contingency Table Between Quintiles of PDI and uPDI**

| **PDI** | **uPDI** | |  | |  |
| --- | --- | --- | --- | --- | --- |
|  | Q1 | Q2 | Q3 | Q4 | Q5 |
| Q1 (*n* = 439) | 99 (22.6) | 100 (22.8) | 84 (19.1) | 68 (15.5) | 88 (20.0) |
| Q2 (*n* = 481) | 97 (20.2) | 99 (20.6) | 99 (20.6) | 93 (19.3) | 93 (19.3) |
| Q3 (*n* = 275) | 75 (27.3) | 51 (18.5) | 64 (23.3) | 36 (13.1) | 49 (17.8) |
| Q4 (*n* = 426) | 99 (23.2) | 88 (20.7) | 90 (21.1) | 70 (16.4) | 79 (18.5) |
| Q5 (*n* = 365) | 70 (19.2) | 88 (24.1) | 72 (19.7) | 63 (17.3) | 72 (19.7) |

**Abbreviations:** PDI: plant-based diet index; uPDI: unhealthful plant-based diet index; Counts (%) are presented within each quintile.

**Supplemental Table 7: Contingency Table Between Quintiles of hPDI and uPDI**

| **hPDI** | **uPDI** | |  | |  |
| --- | --- | --- | --- | --- | --- |
|  | Q1 | Q2 | Q3 | Q4 | Q5 |
| Q1 (*n* = 472) | 63 (13.3) | 93 (19.7) | 101 (21.4) | 88 (18.6) | 127 (26.9) |
| Q2 (*n* = 388) | 79 (20.4) | 86 (22.2) | 73 (18.8) | 54 (13.9) | 96 (24.7) |
| Q3 (*n* = 413) | 101 (24.5) | 83 (20.1) | 88 (21.3) | 72 (17.4) | 69 (16.7) |
| Q4 (*n* = 349) | 82 (23.5) | 73 (20.9) | 79 (22.6) | 61 (17.5) | 54 (15.5) |
| Q5 (*n* = 364) | 115 (31.6) | 91 (25.0) | 68 (18.7) | 55 (15.1) | 35 (9.6) |

**Abbreviations:** hPDI: healthful plant-based diet index; uPDI: unhealthful plant-based diet index; Counts (%) are presented within each quintile.
